# The epidemiological impact of childhood influenza vaccination using live-attenuated influenza vaccine (LAIV) in Germany: predictions of a simulation study

**DOI:** 10.1186/1471-2334-14-40

**Published:** 2014-01-22

**Authors:** Markus A Rose, Oliver Damm, Wolfgang Greiner, Markus Knuf, Peter Wutzler, Johannes G Liese, Hagen Krüger, Ulrich Wahn, Tom Schaberg, Markus Schwehm, Thomas F Kochmann, Martin Eichner

**Affiliations:** 1 Department of Pulmonology, Allergy and Infectious Diseases, Children’s Hospital, Goethe University, Frankfurt, Germany; 2 Department of Health Economics and Health Care Management, Bielefeld School of Public Health, Bielefeld University, Bielefeld, Germany; 3 Department of Children and Adolescents, Dr. Horst Schmidt Klinik, Wiesbaden, Germany; 4 Paediatric Infectious Diseases, University Medicine Mainz, Mainz, Germany; 5 Institute of Virology and Antiviral Therapy, University Hospital, Friedrich Schiller University of Jena, Jena, Germany; 6 Department of Paediatric Infectious Diseases, University Children’s Hospital Würzburg, Würzburg, Germany; 7 Medical Department, AstraZeneca GmbH, Wedel, Germany; 8 Department of Paediatric Pneumology and Immunology, Charité University Medicine, Berlin, Germany; 9 Center of Pneumology, Deaconess Hospital Rotenburg, Rotenburg, Germany; 10 ExploSYS GmbH, Leinfelden-Echterdingen, Germany; 11 Creative-Agents.net e. K, Wallenhorst, Germany; 12 Department of Clinical Epidemiology and Applied Biometry, University of Tübingen, Tübingen, Germany; 13 Epimos GmbH & Co. KG, Uhlandstraße 3, 72144 Dusslingen, Germany

**Keywords:** Influenza, Vaccination, Live-attenuated influenza vaccine, Children, Transmission model, Germany

## Abstract

**Background:**

Routine annual influenza vaccination is primarily recommended for all persons aged 60 and above and for people with underlying chronic conditions in Germany. Other countries have already adopted additional childhood influenza immunisation programmes. The objective of this study is to determine the potential epidemiological impact of implementing paediatric influenza vaccination using intranasally administered live-attenuated influenza vaccine (LAIV) in Germany.

**Methods:**

A deterministic age-structured model is used to simulate the population-level impact of different vaccination strategies on the transmission dynamics of seasonal influenza in Germany. In our base-case analysis, we estimate the effects of adding a LAIV-based immunisation programme targeting children 2 to 17 years of age to the existing influenza vaccination policy. The data used in the model is based on published evidence complemented by expert opinion.

**Results:**

In our model, additional vaccination of children 2 to 17 years of age with LAIV leads to the prevention of 23.9 million influenza infections and nearly 16 million symptomatic influenza cases within 10 years. This reduction in burden of disease is not restricted to children. About one third of all adult cases can indirectly be prevented by LAIV immunisation of children.

**Conclusions:**

Our results demonstrate that vaccinating children 2–17 years of age is likely associated with a significant reduction in the burden of paediatric influenza. Furthermore, annual routine childhood vaccination against seasonal influenza is expected to decrease the incidence of influenza among adults and older people due to indirect effects of herd protection. In summary, our model provides data supporting the introduction of a paediatric influenza immunisation programme in Germany.

## Background

Viral airway infections due to influenza pose severe health problems to humans, critically affecting individuals of any age, especially those with underlying medical conditions. Pandemic and seasonal influenza viruses are a constant public health threat with substantial morbidity and mortality worldwide. There is an increasing body of evidence that documents the health and economic burden of influenza in children [1-3]. Rates of illness are highest among children, and influenza and its complications are responsible for significant healthcare resource use. Especially in young children, hospitalization rates from influenza and its complications are similar to those seen among the elderly population [4-6]. About 40% of all influenza cases, 24% of outpatient visits, 10% of hospitalization days, and 27% of working days lost due to influenza are caused by childhood influenza [7]. In addition, children are the major vectors of influenza transmission within households and communities [8-10], resulting in additional burden of disease from secondary transmission of influenza.

Immunisation provides an effective and efficient tool to control the spread of influenza. Hence, influenza vaccination is widely recommended by international health organizations, mostly targeting the elderly population and people with underlying chronic conditions [11,12]. In 2012, the WHO Strategic Advisory Group of Experts on immunization (SAGE) suggested that children up to 5 years of age should be considered as a target group for annual influenza vaccination [13]. Several national guidelines recommend annual influenza immunisation for children [14]. For instance, since 2010, the US Advisory Committee on Immunization Practices (ACIP) has recommended annual influenza vaccination for all persons aged six months and above [11]. The UK’s Joint Committee on Vaccination and Immunisation (JCVI) which advises the UK government on vaccination policy, recently recommended an extension to the current public influenza immunisation programme to include routine vaccination of children aged 2 to less than 17 years [15]. However, in most countries children do not receive influenza immunisation, although they are highly at risk for infections and tend to spread them through the community [14].

In Europe, immunisation against influenza has been mainly based on injectable trivalent inactivated influenza vaccines (TIV), providing systemic immunity especially in immunocompetent individuals with antecedent contact with the virus. In 2012, intranasally administered live-attenuated influenza vaccine (LAIV) has also become available in Europe, offering superior immunological features and a better protection of children and adolescents than TIV [16-19].

Understanding the modes of influenza transmission and the possible impact of its prevention by vaccines is crucial for physicians, policy makers, and patients in order to better understand the value of influenza immunisation programmes. Based on a complex mathematical model of seasonal influenza transmission and prevention, this paper provides data on the potential population-level impact of different influenza immunisation strategies in Germany. The main objective of this study is to estimate the epidemiological consequences of adding annual routine influenza immunisation for children 2 to 17 years of age using the nasal spray vaccine to the existing German influenza vaccination policy of mostly vaccinating risk-groups.

## Methods

To simulate the transmission of influenza in the German population (82 million inhabitants in 2008), we have developed a deterministic transmission model based on German demographic data using a basic reproduction number (R_0_) of 1.6 [20,21]. Our model considers two influenza strains (influenza A and B) which are transmitted independently without cross-immunity. Transmission dynamics and demographic changes are described by a system of 4,426 differential equations (see Additional file 1). All simulations start on September 1st, 1998 [21]. As the initial age distribution of immunity is unknown, all simulations start with a fraction of 45% immune individuals and are run in with TIV immunisation at current age-specific coverage rates for 14 years before a 10 years lasting evaluation phase begins. During the evaluation phase, TIV immunisation of children is either continued at current coverage rates (scenario 1) or it is replaced by vaccination with LAIV at increased uptake levels (scenario 2) and subsequently the daily differences in influenza infections and symptomatic cases between the two scenarios are calculated. Model parameters are based on published literature or rely on expert opinion elicited by use of the Delphi method. The expert panel consisted of six experts specialised in paediatrics, infectious diseases or pulmonology. All participants of the expert panel were key opinion leaders in their respective research areas and were identified through relevant publications, involvement in influenza-related research activities or research networks on infectious respiratory diseases. Expert opinion was elicited using a two round Delphi study and a final consensus meeting. The questionnaires included questions on influenza transmission dynamics, the course of disease and German-specific treatment patterns. At the beginning of the Delphi process, all panellists were provided with the results of a systematic literature search on relevant parameters. After each round, the experts were provided with a summary of the experts’ responses. The response rate for both rounds was 100%. Finally, the results of the Delphi survey were discussed with all experts at a consensus meeting. When a consensus could not be reached among all panellists, mean or median values of the experts’ estimates were calculated. Ethical approval was not required for this study. Details of the simulation model are as follows.

### Transmission model

**Figure 1:**
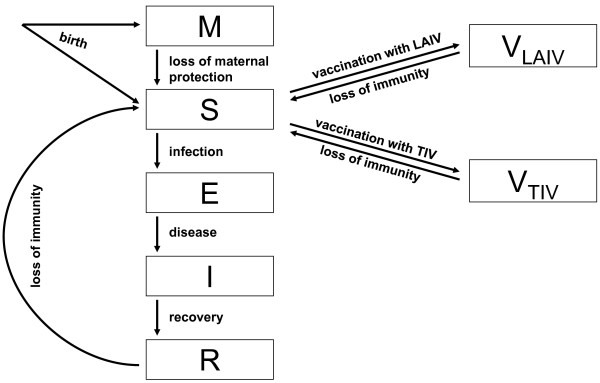
**Model structure.** Individuals are either born susceptible (S) or with maternal protection (M), which prevents infection and successful vaccination. Only susceptible individuals can successfully be vaccinated (V_LAIV_ and V_TIV_) or infected. When infected, they pass through a latent period (E) before becoming infectious (I) and finally become immune (R). Individuals can completely lose their immunity (derived by infection or vaccination) and become susceptible again.

The model extends the general structure of the classic SEIRS (susceptible-exposed-infectious-recovered-susceptible) model by adding the state of maternal protection (M) and two different classes of vaccine-induced immunity (V_LAIV_ and V_TIV_) (see Figure 1). Adults are further grouped into age cohorts and risk classes. Children and adolescents are classified into several age groups without risk differentiation. A fraction of 30% of all newborn individuals is assumed to be protected by maternal antibodies for an average duration of four months during which they cannot be infected. After losing their maternal protection, children become susceptible (S) and can successfully be vaccinated or infected. If they become infected, they first pass through a latent period (E) which lasts on average one day before they become contagious (I). Following this latent phase, they recover on average after 5 days and become immune [22]. Naturally acquired immunity lasts on average for 6 years after infection with influenza A and for 12 years after infection with influenza B. These durations account for waning memory of the immune system and the accumulation of antigenic changes in the pathogens. In our transmission model, we use the German POLYMOD contact matrix which describes the mixing of the age groups [23]. The transmission probability per contact is calculated such that the largest eigenvalue of the next generation matrix yields a basic reproduction number R_0_ = 1.6. Following the recommendations of the expert panel, we have used a value of 1.6 for the basic reproduction number. This number lies within the range estimated as the variation across influenza seasons and countries by Chowell et al. [20] (0.9-2.1). Chowell et al. [20] have published a confidence interval that ranges from 1.2 to 1.4. As we address both influenza A subtypes as “strain A” and both influenza B lineages as “strain B”, we have to use a higher value for the basic reproduction number of these “strains” than what would be needed for four (or even more) independently transmitted virus types. The choice of R_0_ = 1.6 led to a good fit to observed influenza incidence (see model validation section). The resulting contact rate is varied seasonally with a peak around Christmas which is 43% higher than the baseline [21]. It is further assumed that the whole population is exposed to infection from abroad at a low constant “external infection rate” which yields about 1 infection per 1,000 susceptible person years. A total of 66.9% of all infected individuals develop symptoms [22]; their symptom state is assumed not to alter the transmissibility of infection.

### Demographic model

The population is structured in 96 age classes of one year each (except for the last age class which contains all individuals who are 95 years of age or older). People die according to age-specific rates which were derived from German 2008 data [24]. In order to keep these parameters constant for all simulation years, we used the 2008 demographic distribution and mortality rates to back-calculate the demographic distribution and birth rates of earlier years (see Additional file 1 for details). The annual numbers of births in later years were obtained by linear extrapolation, using the values of previous years. In the simulation model, births and deaths occur throughout the year, but ageing steps are only performed at the end of each simulation year, i.e. on September 1st, mimicking the transition in German school classes (e.g. “11 year old children” become “12 year old children” on September 1st of each year) [21]. Adults are further subdivided into people with “normal risk” and people with “increased risk” due to underlying chronic disease (see Table 1) [25].

**Table 1:** Parameters of the transmission model

**Parameter**	**Description**	**Base-case value**	**Reference**
*R*_0_	Basic reproduction number	1.6	Chowell et al. [20]; expert opinion
z	Amplitude of seasonal transmission	43%	Vynnycky et al. [21]
*α*	Outside infection rate (per person per year)	0.001	Assumption
*D*_*L*_	Average duration of the latent period (days)	1	Carrat et al. [22]
*D*_*I*_	Average duration of the infectious period (days)	5	Carrat et al. [22]
*f*_*K*_	Symptomatic fraction of infected individuals	66.9%	Carrat et al. [22]
*f*_*med*_	Fraction of symptomatic cases who seek medical help (i.e. physician consultation)		
	- children below 2 years of age	60%	Expert opinion
	- children from 2 to 6 years of age	40%	Expert opinion
	- children from 7 to 12 years of age	30%	Expert opinion
	- juveniles from 13 to 17 years of age	10%	Expert opinion
	- adults below 60 years of age without increased risk	20%	Expert opinion
	- adults with increased risk or above 60 years of age	50%	Expert opinion
*f*_*R*_	Immune fraction before initialising the simulations	45%	Assumption
*m*	Fraction of newborns protected by maternal antibodies	30%	Assumption
*D*_*M*_	Average duration of maternal protection (months)	4	Expert opinion
$D_{R}^{\left(A\right)}$	Average duration of naturally acquired immunity to influenza A (years)	6	Vynnycky et al. [21]
$D_{R}^{\left(B\right)}$	Average duration of naturally acquired immunity to influenza B (years)	12	Vynnycky et al. [21]
*D*_*TIV*_	Average duration of TIV induced immunity (years)	0.7	Assumption
*D*_*LAIV*_	Average duration of LAIV induced immunity (years)	2.8	Guided by Tam et al. [26]
*r*_18–44_	Percentage of people from 18 to 44 years of age with elevated risk	7.6%	Fleming & Elliott [25]
*r*_45–59_	Percentage of people from 45 to 59 years of age with elevated risk	17.6%	Fleming & Elliott [25]

*TIV* trivalent inactivated influenza vaccine; *LAIV* live-attenuated influenza vaccine.

### Vaccination model

Vaccinations are performed annually from October 1st to November 30th (official German recommendation), whereby a constant number of individuals are vaccinated on each day. Each individual can be only vaccinated once per year, receiving either TIV or LAIV. Depending on the type of vaccine and on the age and risk status of the vaccinated individuals, a fraction of susceptible individuals becomes temporarily immune after vaccination (see Table 2). The duration of vaccination-derived immunity depends on the type of vaccine: based on the vaccine efficacy measured in the first and second transmission season, an average duration of 2.8 years of immunity has been calculated for LAIV [18,19,26], whereas TIV-induced immunity is assumed to be lost on average after 0.7 years [19,27-29] (details for the calculations are given in the Additional file 1).

**Table 2:** Vaccination parameters

**Age class**	**Vaccine efficacy**^**a**^		**Reference**	**Vaccination coverage**			**Reference (initial value)**
				**Scenario 1**	**Scenario 2**		
	**TIV**	**LAIV**		**TIV**	**TIV**	**LAIV**	
1 year	11.0%	N/A	Vesikari et al. [41]	19.2%	19.2%	---	Blank et al. [30]
2 years	59.0%	80.0%	Jefferson et al. [19]; Rohrer et al. [18]	19.2%	19.2%	22.4-50%^b^	Blank et al. [30]
3-6 years	59.0%	80.0%	Jefferson et al. [19]; Rohrer et al. [18]	22.4%	N/A	22.4-50%^b^	Blank et al. [30]
7-10 years	59.0%	80.0%	Jefferson et al. [19]; Rohrer et al. [18]	23.6%	N/A	23.6-50%^b^	Blank et al. [30]
11-17 years	59.0%	80.0%	Jefferson et al. [19]; Rohrer et al. [18]	11.0%	N/A	11.0-50%^b^	Blank et al. [30]
18-59 years, normal risk	68.0%	N/A	Monto et al. [29]	14.5%	14.5%	N/A	Blank et al. [30]
18-59 years, elevated risk	58.0%	N/A	Jefferson et al. [28]	29.8%	29.8%	N/A	Blank et al. [31]
60-64 years	58.0%	N/A	Jefferson et al. [28]	33.1%	33.1%	N/A	Blank et al. [31]
65-69 years	58.0%	N/A	Jefferson et al. [28]	47.6%	47.6%	N/A	Blank et al. [31]
70 years and over	58.0%	N/A	Jefferson et al. [28]	53.4%	53.4%	N/A	Blank et al. [31]

*TIV* trivalent inactivated influenza vaccine; *LAIV* live-attenuated influenza vaccine; *N/A* not applicable.

^a^Vaccine efficacy determines the fraction of (previously susceptible) vaccinees who are immune due to vaccination at the peak of the following transmission season (i.e. 100 days after vaccination).

^b^LAIV coverage increases in three annual steps from the initial to the final percentage.

### Vaccination scenarios

During the 14-years lasting run-in phase of the simulations, only TIV is used. In the subsequent intervention phase, the initial age-dependent administration of TIV is either continued unchanged (scenario 1) or vaccination of children from 2 to 17 years of age is replaced by immunisation with LAIV (scenario 2) whereby the vaccination coverage increases from the baseline value up to 50% in three annual steps [30,31].

### Model validation

We validated our simulation model in four ways: First, the simulated age distributions in the years 2010, 2015, and 2020 are compared to demographic predictions of the German Federal Statistical Office [32]. Second, the incidence of infections of children and young adults is compared with published observations [33,34]. Third, the simulated number of physician visits due to influenza is compared to influenza-attributed excess consultations published by the Robert Koch Institute (RKI) [35]. Based on the results of the Delphi panel, we assume that the following percentages of infected individuals seek medical care: 60% of children younger than 2 years of age, 40% of children from 2 to 6 years, 30% of children from seven to 12 years, 10% of juveniles (13 to 17 years), 20% of otherwise healthy adults from 18 to 59 years, and 50% of elderly and adults with increased risk. Using the same age classes as the RKI (i.e. 0–4, 5–14, 15–34, 35–59, 60+), we calculate the annual number of outpatient visits per 100,000 individuals for each year of the second half of our initialization period (i.e., for the years 2004 to 2011). Fourth, in order to check for coding errors, the transmission model was coded twice by two software developers who coded the simulations independently: one software developer used JAVA and solved the differential equation system numerically, using the fourth-order Runge–Kutta method with automatic step size control [36]. The other software developer used the programming languages C and Scilab version 5.3 (an open-source analogue of MatLab), employing the hybrid ODE solver “rkm9mkn” (Intel® Ordinary Differential Equation Solver Library) which automatically chooses between an implicit and an explicit method, depending on the results of a repetitive calculation of the Jacobi matrix of the differential equation system. In the final validation, the resulting annual and total numbers of infections and vaccinations were compared.

## Results

### Validation results

The validation demonstrated good model fit in various areas. The estimated incidence of infections of young adults matched the published observations. Williams et al. [33] showed that 10.6% of examined health care workers and controls had a serologically confirmed influenza infection or an at least fourfold rise in influenza antibody titre during the 2006/2007 influenza season in Germany. In our simulation study, 4,098,465 infections occur in the group of young healthy adults (18 to 59 years without increased risk) in the same time period. Relating these infections to the group size of 42,284,307 individuals yields a simulated infection rate of 9.7% which is similar to the observation of Williams et al. [33]. Using the set of parameter values from Chowell et al. [20] (R_p_ = 1.3; duration of the latent period = 1.9 days; duration of the infectious period = 4.1 days) instead of our set of parameters would lead to an infection incidence of 15.56% per year among young adults. Likewise, using the set of parameter values from Pitman et al. [37] (R_0_ = 1.8; duration of the latent period = 2 days; duration of the infectious period = 2 days) would lead to an infection incidence of 12.69% per year for young adults which is quite a bit higher than the observed infection incidence in Germany.

Williams et al. [33] furthermore report that about 30% of individuals with serologically confirmed influenza infection did not report influenza-like symptoms, which corresponds well to the 33.1% of asymptomatic infections which is assumed in our simulation study.

Since German data on childhood influenza infection incidence is scarce, we used estimates from an international systematic review [34] to validate our simulation results for children. Bueving et al. [34] included 28 studies and found a wide variation in the incidence of laboratory-proven influenza illness ranging from 0 to 46% in children aged 0–19 years. However, only two of the included studies were based on long-term observations. These studies reported an average seasonal incidence of 4.6% in children aged 0–19 years and 9.5% in children < 5 years. Our model simulates an influenza illness incidence of 5.57% in children aged 0–19 years.

**Figure 2:**
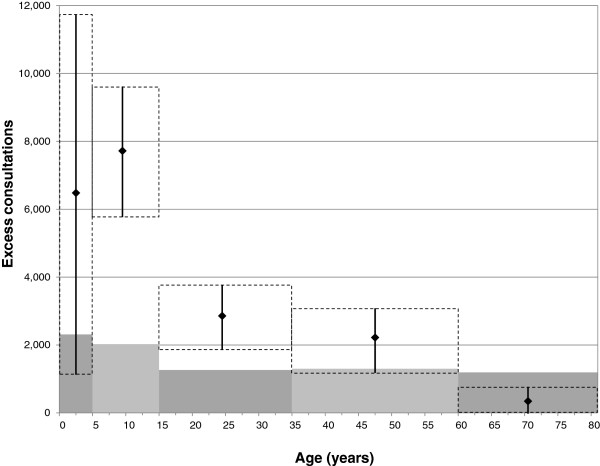
**Excess consultations per 100,000 per year.** Grey bars represent outpatient physician visits simulated by the model, dots with 95% confidence intervals are estimates of influenza-associated excess consultations published by the RKI.

Figure 2 compares the simulated number of physician visits before the introduction of LAIV immunisation to data on estimated influenza-associated excess consultations published by the RKI [34]. Except for the age class of the elderly, our simulation results are much lower than the values reported by RKI. Our simulated age distribution corresponds very well to the official German predictions, only the number of children and some of the age classes of elderly are slightly lower in our simulations than what is officially predicted (see Additional file 1 for details).

The results of the internal model validation, using two independent programming approaches, showed a nearly perfect concordance of the simulation results obtained with the two different software systems: for the chosen set of parameters (which differed slightly from the parameters used in this publication), a total of about 96,500,000 infections occurred in scenario 1 in the total simulation time of 24 years, yet the two programmes differed by less than 0.2 infections. Likewise, the total number of vaccinations deviated by less than a millionth of a per cent.

### Base-case analysis

**Figure 3:**
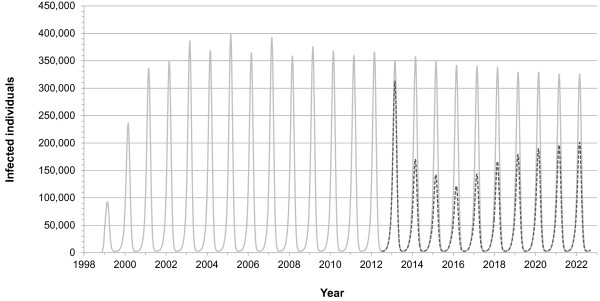
**Simulated seasonal fluctuation in influenza infections.** The light grey solid curve shows influenza A and B infections in scenario 1 where only TIV is used, the dark grey dashed curve shows those in scenario 2 where TIV immunisation of children 2 to 17 years old is replaced in 2012 by LAIV immunisation and where childhood vaccination coverage with LAIV is subsequently increased up to 50% in three annual steps.

**Figure 4:**
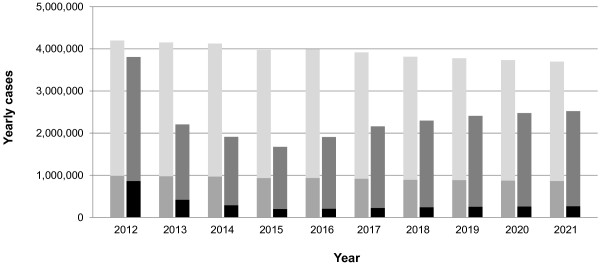
**Annual average number of symptomatic influenza cases.** The left bar of each pair shows symptomatic influenza cases in scenario 1 where only TIV is used, the right bar of each pair represents those in scenario 2 where TIV immunisation of children 2 to 17 years old is replaced in 2012 by LAIV immunisation and where childhood vaccination coverage with LAIV is subsequently increased up to 50% in three annual steps. The lower parts of the bars correspond to the number of paediatric cases, the upper parts of the bars indicate the number of cases in adults aged 18 years and above.

Figure 3 shows the simulated dynamics of symptomatic influenza cases. Due to the seasonal fluctuations in the transmissibility of influenza and annual vaccination campaigns, influenza transmission shows an expressed seasonality with peaks at the end of February. During the run-in phase, a percentage of children and adults receive annual vaccination with TIV (scenario 1; light grey solid curve). In the autumn of 2012, TIV immunisation of children from 2 to 17 years is fully replaced by LAIV immunisation whereby the vaccination coverage is increased in three annual steps up to its final value of 50% (scenario 2; dark grey dashed curve). Figure 4 shows the simulated total annual number of symptomatic influenza cases in the 10-year evaluation phase. The left bar of each pair shows the results of scenario 1 with TIV immunisation, the right bar of each pair shows those of scenario 2 with LAIV immunisation of children. Whereas cases decline only moderately in scenario 1, they drop considerably in the first years of scenario 2 and increase again thereafter.

During the 10-year evaluation phase, a total of 58.9 million infections occur in scenario 1 (37.9 million influenza A infections, and 20.9 million influenza B infections). A total of 23.9 million infections are prevented by additional LAIV immunisation (13.7 million A, and 10.2 million B) in scenario 2. Table 3 shows that nearly two thirds of infections and symptomatic cases of children and juveniles are prevented by LAIV vaccination. This reduction is not restricted to children: about one third of all adult cases can indirectly be prevented by LAIV immunisation of children. Overall, LAIV vaccination prevents more than 40% of all influenza infections and symptomatic cases in the German population. When relating the number of 23.9 million prevented infections and 16 million prevented symptomatic cases to the additional 27.4 million vaccinations in scenario 2, it shows that on average 1.15 vaccinations prevent one infection, or that 1.71 vaccinations prevent one symptomatic case (number needed to vaccinate, NNV).

**Table 3:** Simulated numbers of influenza vaccinations and outcomes in children and adults using base-case values

**Vaccinations and outcomes**^**a**^	**Children (0–17 years)**			**Adults (18 years and over)**		
	**Scenario 1**^**b**^	**Scenario 2**^**c**^	**Difference**	**Scenario 1**^**b**^	**Scenario 2**^**c**^	**Difference**
**Vaccinations**						
TIV	19,297,651	1,084,672	−18,212,979	180,955,563	180,955,563	0
LAIV	0	45,637,434	45,637,434	0	0	0
Total	19,297,651	46,722,106	27,424,455	180,955,563	180,955,563	0
**Epidemiological outcomes**						
Infections	13,830,361	4,835,746	−8,994,615 (−65.0%)	45,033,115	30,122,649	−14,910,466 (−33.1%)
Symptomatic cases	9,252,511	3,235,114	−6,017,397 (−65.0%)	30,127,153	20,152,052	−9,975,101 (−33.1%)

*TIV* trivalent inactivated influenza vaccine; *LAIV* live-attenuated influenza vaccine.

^a^All results are estimates for the 10-year evaluation period.

^b^Scenario 1: TIV immunisation at current age-specific vaccination coverage.

^c^Scenario 2: TIV immunisation of children 2 to 17 years of age is replaced by LAIV immunisation, with coverage increasing up to 50% in three annual steps.

### Sensitivity analyses

**Figure 5:**
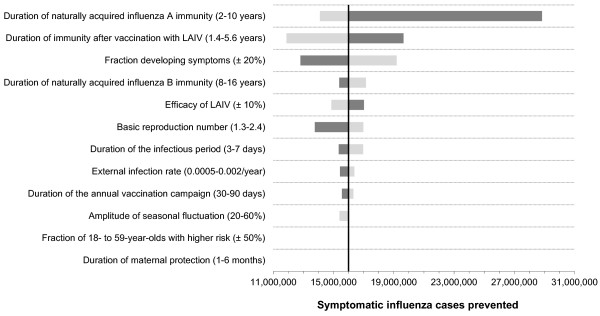
**Results of one-way sensitivity analyses.** Each of the horizontal bars of this tornado chart shows the impact of varying a single parameter of the model across a given range on the number of symptomatic influenza cases prevented while keeping all other parameters at their base values. The dark grey bars represent the upper bound of the range, the light grey bars represent the lower bound. The prevented cases are the difference of symptomatic influenza cases between scenario 1 and scenario 2 during the 10-year evaluation period. TIV is used constantly in scenario 1, whereas TIV immunisation in children 2 to 17 years of age is replaced by LAIV immunisation in scenario 2, with coverage increasing up to 50% in three annual steps.

In our base-case analysis, LAIV-induced immunity is assumed to last much longer than TIV-induced immunity. When halving the duration of LAIV-induced immunity, the number of prevented symptomatic influenza cases decreases from 16 to 12 million within 10 years. Figure 5 compiles results of univariate sensitivity analyses where key parameter values are modified individually whereas all remaining parameter values are kept unchanged. The fraction of infected individuals who become symptomatic is a parameter with a very high influence on the difference of symptomatic cases between scenario 1 and 2. The duration of naturally acquired influenza A immunity is also very influential. Due to the dominance of influenza A cases, it has much more impact than the duration of influenza B immunity. The basic reproduction number determines the number of secondary cases and the duration of the infectious period determines the spread of the infection and, thus, these parameters have a major impact on the results. The vaccine efficacy of LAIV complements the list of most influential factors.

**Figure 6:**
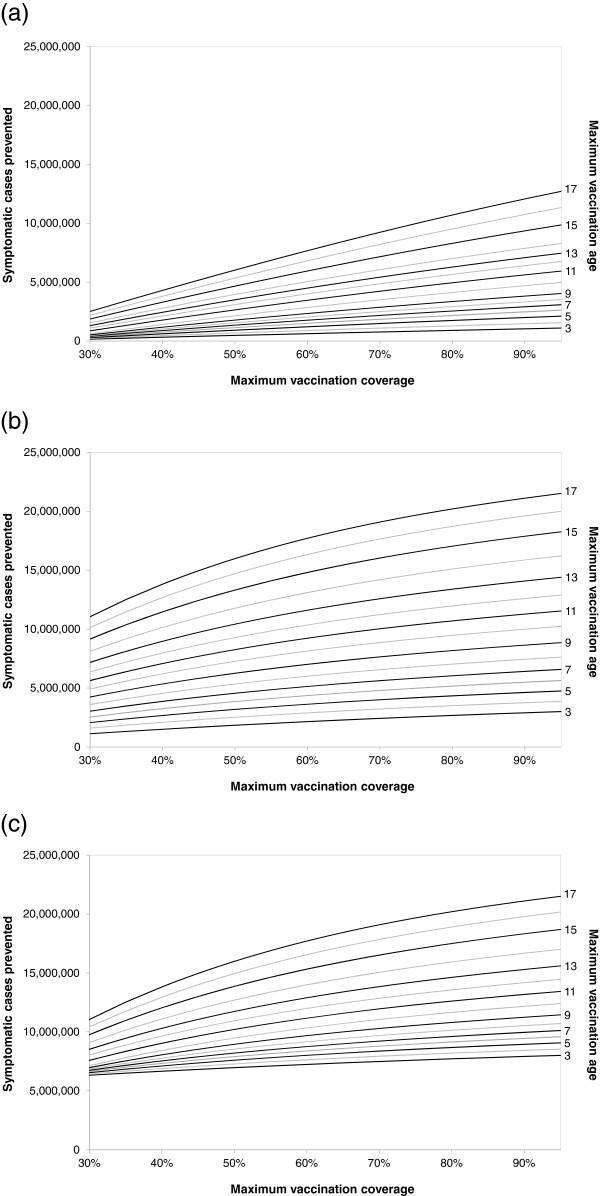
**Results of two-way sensitivity analyses varying the vaccination coverage and the maximum vaccination age.** These charts show how many additional symptomatic influenza cases are prevented in scenario 2 during the 10-year evaluation period in Germany when compared to scenario 1. In scenario 1, TIV is used for all age classes with constant age-specific vaccination coverage, as reported for Germany. Scenario 1 remains unchanged in all analyses presented by these graphs. Scenario 2 assumes that annual vaccination of children up to a given maximum age (see numbers on the right hand side) is recommended in Germany, starting in 2012. In scenario 2, the vaccination coverage of children from 2 years up to the recommended age is increased in three annual steps, starting from the baseline value and finally reaching the coverage given on the horizontal axis; the vaccination coverage of children in other age groups and of adults is kept at the baseline value (which is also used in scenario 1). **(a)** TIV is used for all children and adults in scenario 2; **(b)** LAIV is used for all children from 2 years up to the recommended maximum age of childhood vaccination, and TIV is used for all others; **(c)** LAIV is used for all children from 2 years up to the recommended maximum age of childhood vaccination (with increasing coverage) and for all older children up to 17 years (with constant coverage); TIV is used for all others.

In the base-case analysis, we have assumed that it will be recommended to vaccinate children between 2 and 17 years of age with LAIV and that these children will gradually increase their annual vaccination coverage while children under 2 years of age continue to receive TIV at the same level of uptake. The recommended vaccination scheme starts in 2012 with children receiving either TIV or LAIV at the baseline coverage shown in Table 2. In the following three years, LAIV coverage increases up to 50%. In Figure 6, we modify many of these assumptions: we change the maximum vaccination age for children from 17 years to another age (shown at the right hand side of the curves). We further assume that the final vaccination coverage of these children will not be 50%, but a value between 30 and 95% (given on the horizontal axis). In Figure 6a, we assume that all children receive TIV; children up to the maximum recommended age increase their vaccination coverage; older children are constantly vaccinated with the same coverage as in scenario 1. In Figure 6b, we assume that children below 2 years of age receive TIV with increasing coverage, children from 2 years to the maximum recommended age receive LAIV with increasing coverage, and older children receive TIV (at constant scenario 1 vaccination coverage). In Figure 6c, we assume that children below 2 years of age receive TIV with increasing coverage, children from 2 years to the maximum recommended age receive LAIV with increasing coverage, and older children receive LAIV, but at the same constant coverage as is used for TIV in scenario 1. All these changes are made in scenario 2, whereas scenario 1 always remains unchanged.

On the vertical axes, Figure 6 shows how many symptomatic influenza cases would be prevented by the vaccination strategy described in scenario 2 as compared to scenario 1 during the 10 year evaluation period. The maximum number of prevented symptomatic cases is always reached if 95% of all children up to 17 years of age are vaccinated annually. In Figure 6a (only TIV vaccination), scenario 2 would prevent 12.7 million more symptomatic cases in 10 years than scenario 1 would; in Figure 6b (LAIV and TIV vaccination) and 6c (only LAIV vaccination for children from 2 years of age), scenario 2 would prevent 21.5 million more symptomatic cases in 10 years than scenario 1 would. The vaccination strategy of using only LAIV (Figure 6b) in older children can only differ from the mixed strategy (Figure 6c) if the maximum vaccination age is below 17 years. In that case, more cases are prevented by the “only LAIV” strategy than by the mixed strategy, especially, if the annual vaccination coverage is low. Both LAIV strategies prevent more symptomatic cases than the strategy which uses only TIV, most noticeably for low annual vaccination coverage.

We also developed an alternative model that totally ignores herd immunity effects. When using this model, the overall reduction in symptomatic cases induced by LAIV drops from 40% to 10%. This shows that the indirect protection accounts for a large part of the total benefit.

## Discussion

We have presented the results of several simulations which were produced by a dynamic transmission model described by more than 4,000 differential equations. Even though our model considers the most important demographic and epidemiologic features of seasonal influenza transmission, every model – irrespective of its complexity – must be regarded as a gross simplification of reality.

Below we discuss our methodological decisions. Our model considers age-dependent contact patterns, but does not consider spatial or social structures. The model assumes that the frequency of contacts between individuals only depends on their age. We use the age distribution for Germany with birth rates consistent with published data and age-dependent mortality rates, but we ignore changes of the population caused by migration. We update the age of individuals only once every year after the end of summer, as is done in other models of influenza transmission and vaccination [21,37].

In our vaccination model, age-dependent fractions of the population are either vaccinated with TIV or with LAIV during October and November. However, we do not keep track of who has received which type of vaccine in previous years or who experienced an infection before vaccination. Consequently, we apply vaccinations independently of each other and use average values for the age-dependent vaccine efficacy which does not consider pre-existing immunity. As LAIV vaccination has not been approved for children below 2 years of age, they only receive TIV in all simulation scenarios. Due to the classification of individuals in 1-year age classes and because of the annual ageing step at the end of summer, we omit vaccination of newborn children between six and 12 months of age in both simulation scenarios, which resulted in a slight underestimation of the beneficial effect of childhood vaccination.

The variability of the viral strains over time is considered in our model only indirectly by assuming that it takes on average six years until an individual can be infected again with influenza A; protection against influenza B lasts on average twelve years [21], as B strains tend to be more stable than A strains. We assume that 66.9% of infections lead to symptoms, but – due to the lack of solid information – we have not implemented a dynamically changing fraction of partially immune individuals who may have a milder course of disease or a reduced contagiousness.

Our simulation results show the typical seasonal waves which usually peak at the end of February (Figure 3). Without additional childhood vaccination, our simulations lead to about four million symptomatic cases per year in Germany (Figure 4). About one third of all simulated symptomatic cases in scenario 1 (35.6%) are caused by influenza B.

The conservative character of our approach is reflected by the number of simulated medical consultations being lower than the data published by the RKI (Figure 2). Solid data on the age-specific infection incidence in Germany are scarce, but we were able to compare our simulation results to the infection incidence among young adults [33], obtaining a good concordance. The same applies to the clinical attack rate in children when compared to estimates from international studies. Adding the agreement of our double-coding results, this indicates that our model is reliable and trustworthy.

Some simulation parameters are not precisely known, while others may change from season to season due to changes in the viral strains. We have addressed this insecurity by various sensitivity analyses. As our main outcome is the difference between the number of symptomatic cases in the scenarios with and without additional childhood vaccination, the fraction of infections which leads to symptoms is one of the most influential parameters (Figure 5). Varying the duration of vaccine-induced protection of LAIV has also a large impact on the results. Hence, there is a great need for future research on this subject.

Another important parameter is the duration of immunity after the infection (most notably for influenza A which causes two thirds of cases): if immunity lasts longer, fewer cases occur and fewer cases can be prevented by childhood vaccination. For the same reason, the basic reproduction number R_0_ which determines the transmissibility of influenza also strongly influences the results. As changes in the basic reproduction number can result in bi-annual cycles with consecutive large and small seasonal waves, it is somewhat difficult to predict whether a modification of the value of R_0_ leads to larger or smaller numbers of prevented cases.

Various simulation studies have assessed the clinical and economic implications of childhood vaccination against seasonal influenza in different settings. These evaluations have shown that influenza vaccination of children is cost-effective and may even be cost-saving [38,39]. However, most of the previous simulation studies have lacked consideration of herd immunity effects. Results of recently published studies based on dynamic transmission models underline the importance of taking indirect protection (herd immunity) benefits to the community into account [37,40]. Our model results confirm these findings.

## Conclusions

Paediatric influenza represents a substantial burden to public health. Children have high influenza illness rates and transmit the disease within the community, posing considerable health and financial consequences to the society. In this paper, we evaluated the epidemiological impact of the implementation of an additional LAIV-based childhood influenza immunisation programme in Germany. Our results demonstrate that vaccinating children 2–17 years of age is likely associated with a significant reduction in the burden of paediatric influenza. Furthermore, annual routine childhood vaccination against seasonal influenza is expected to decrease the incidence of influenza among adults and older people due to indirect effects of herd protection. These results will be used to inform a cost-effectiveness analysis of universal childhood influenza vaccination in Germany.

In summary, our model provides data supporting the implementation of a paediatric influenza immunisation programme in Germany. Due to the high efficacy and the easy and painless route of administration, the use of LAIV in children aged 2–17 years could be an important part of such a programme.

## Competing interests

MAR has received research support, speaking fees and honoraria for attending advisory boards from AstraZeneca, SPMSD, and Novartis Vaccine.

OD has conducted studies for and received honoraria from herescon GmbH, which has received research support and consulting fees from AstraZeneca and MedImmune.

WG is shareholder of herescon GmbH, a contract research and consulting institute, which has received research support and consulting fees from AstraZeneca and MedImmune.

MK received honoraria and consulting fees for presentations, publications and participation in advisory boards from Astra Zeneca and MedImmune.

PW has received honoraria for consulting from AstraZeneca and honoraria for attending advisory boards from AstraZeneca, MedImmune and Sanofi Pasteur MSD. Furthermore, PW has received honoraria for speaking at scientific symposia from AstraZeneca and Berlin-Chemie.

JGL has received honoraria for consulting, attending advisory boards and speaking at scientific symposia from AstraZeneca.

HK is an employee of AstraZeneca and as such receives a salary from the company.

UW has received honoraria for consulting and attending advisory boards from AstraZeneca.

TS has received honoraria for attending advisory boards from AstraZeneca, MedImmune, GlaxoSmithKline, Pfizer and Novartis. Furthermore, TS has received honoraria for speaking at scientific symposia from AstraZeneca, GlaxoSmithKline, Pfizer and Novartis.

MS is employee and shareholder of ExploSYS GmbH, which has received payments from Epimos GmbH & Co. KG, a contract research and consulting institute, which has received research support and consulting fees from AstraZeneca.

TFK declares that he does not have financial or non-financial competing interests.

ME is partner and shareholder of the contract research and consulting institutes Epimos GmbH & Co. KG and Epimos Beteiligungs-GmbH,respectively, which received consulting fees or research support from AstraZeneca, Novartis and GlaxoSmithKline.

## Authors’ contributions

ME and OD conceptualised the study, performed data collection and carried out the simulations. ME and OD also interpreted the results and drafted the manuscript. MAR participated in the interpretation of data and helped to draft the manuscript. MS provided technical support and programmed the model in Java. TFK programmed the model using C and Scilab. All authors contributed to the critical revision of the initial draft and approved the final version of the manuscript.

## Pre-publication history

The pre-publication history for this paper can be accessed here:

http://www.biomedcentral.com/1471-2334/14/40/prepub

## Supplementary Material

Additional file 1 This appendix provides detailed explanations of the model including equations. Click here for file
