# Supplementary material for: The epidemiological impact of childhood influenza vaccination using live-attenuated influenza vaccine (LAIV) in Germany: predictions of a simulation study
Source: BMC Infect Dis. 2014 Jan 22;14:40. doi: 10.1186/1471-2334-14-40 (PMC3905925; doi:10.1186/1471-2334-14-40)
Supplement: Additional file 1 — This appendix provides detailed explanations of the model including equations. [file 1471-2334-14-40-S1.pdf]

The epidemiological impact of childhood influenza vaccination using live attenuated influenza vaccine (LAIV) in Germany: predictions of a simulation study

Markus A. Rose; Oliver Damm; Wolfgang Greiner; Markus Knuf; Peter Wutzler; Johannes G. Liese; Hagen Krüger; Ulrich Wahn; Tom Schaberg; Markus Schwehm; Thomas F. Kochmann; Martin Eichner

SUPPLEMENTARY APPENDIX

To simulate the transmission of influenza in the German population, we have developed a simulation model based on 4,426 differential equations which is described in detail in this document. All simulations start on September 1st, 1998 (simulation years always start on September 1st of each year to mimic the German school year). They are run in with either inactivated vaccine (TIV) vaccination for 14 years. During the evaluation phase which starts in 2012, TIV vaccination (scenario 1) is replaced by LAIV vaccination for children of 2 years and older (scenario 2) and the daily differences between the two scenarios are calculated.

1 Demography

Age cohorts

The population is structured in 1-year age classes. Births and deaths occur throughout the year, but ageing steps (e.g., 11 year old children become 12 year old children) are only performed at the end of each simulation year, i.e., on September 1st. From demographic databases, the numbers of individuals $N_a^{(2008)}$ who were of age a at the end of the year 2008 are given for ages 0 to 84 years. Additionally, the total number of individuals who are at least 85 years old was given (these were split up into cohorts of one year each as described below). Although the demographic data were reported for December 31st, 2008, they were assumed to reflect the situation in Germany at the end of August 31st,2008, i.e., immediately before the 2008 simulation year which starts on September 1st, 2008.

Age-dependent mortality

For individuals up to 70 years of age, the mortality per year d_a is the average value of the age-dependent fractions of men and women who died in 2008 (see Table S1). For individuals over 70 years, such data were not available. For the age groups from 70 to 83 years, average mortality values of 2004 to 2007 were used instead. For individuals over 83 years, we assumed exponential survival (i.e. a constant fraction $1-d_{\geq 84}$ survives one year, irrespective of age). With this assumption, the $N_{\geq 85}^{(2008)}$= 1,802,376 individuals who are at least 85 years old could be split up into annual cohorts by using the relationship $N_{84}^{(2008)} + N_{\geq 85}^{(2008)} = \sum_{i=0}^{\infty} N_{84+i}^{(2008)} = \sum_{i=0}^{\infty} N_{84}^{(2008)}\left(1-d_{\geq 84}\right)^i = \frac{N_{84}^{(2008)}}{d_{\geq 84}}$ for all ages $a \geq 84$. Solving this equation yielded $d_{\geq 84} = \frac{N_{84}^{(2008)}}{N_{84}^{(2008)} + N_{\geq 85}^{(2008)}}$. From all fractions d_a, daily mortality rates were calculated as $\sigma_a = -\ln\left(1-d_a\right)/365$ which were used in the differential equations.

Table S1. Demography of Germany in 2008 (based on Statistisches Bundesamt 2010).

age class a	number of individuals in 2008 $N_a^{(2008)}$	mortality per year d_a	age class a	number of individuals in 2008 $N_a^{(2008)}$	mortality per year d_a
0	683350	0.003736975	43	1433621	0.001543930
1	688205	0.000309925	44	1458688	0.001695345
2	676795	0.000183330	45	1450926	0.001969045
3	689055	0.000149460	46	1406185	0.002217255
4	707767	0.000124885	47	1385462	0.002490415
5	708961	0.000104195	48	1347055	0.002739995
6	721364	0.000087990	49	1304418	0.003070175
7	738257	0.000085610	50	1237602	0.003384935
8	771931	0.000087155	51	1211829	0.003716400
9	774484	0.000080055	52	1181955	0.004104020
10	790980	0.000079195	53	1147173	0.004429365
11	816759	0.000086510	54	1127912	0.004832700
12	802868	0.000099335	55	1092617	0.005240435
13	778980	0.000106620	56	1094070	0.005685915
14	789350	0.000134160	57	1071281	0.006061620
15	821725	0.000168315	58	1076343	0.006733895
16	844154	0.000228190	59	1037905	0.007295860
17	878572	0.000263130	60	951651	0.007906060
18	969220	0.000401255	61	894146	0.008675950
19	965959	0.000412350	62	780604	0.009216185
20	1002389	0.000406995	63	689175	0.010101415
21	989509	0.000402270	64	913155	0.010718705
22	982397	0.000413960	65	925168	0.011535380
23	960436	0.000413705	66	897432	0.012672945
24	965108	0.000396465	67	1082064	0.013588340
25	978716	0.000412000	68	1134524	0.015022165
26	1008347	0.000446820	69	1105369	0.016448290
27	1007662	0.000427855	70	1023231	0.018151840
28	1018060	0.000430225	71	945240	0.021241785
29	971407	0.000458850	72	907323	0.024461856
30	959710	0.000468875	73	860695	0.026592360
31	952482	0.000515370	74	785767	0.030186033
32	940425	0.000530620	75	620088	0.033463025
33	917670	0.000565745	76	602648	0.036786835
34	932383	0.000603115	77	598135	0.041129041
35	943717	0.000655395	78	609940	0.045495200
36	1036781	0.000704695	79	570277	0.050703805
37	1145602	0.000776695	80	542723	0.056091309
38	1199102	0.000851815	81	484125	0.063143855
39	1288341	0.000977000	82	450513	0.070516749
40	1350555	0.001080750	83	418497	0.079220446
41	1385779	0.001195305	84	362878	0.167591423
42	1424256	0.001358540	≥ 85	1802376	0.167591423

Back-calculated age-distribution of 1998

In order to initialize the epidemiologic and immunologic age distributions, our simulations were run in for 14 years, starting on September 1st, 1998. As we kept the age-dependent mortality rates σ_a constant throughout all simulation years, we had to obtain the age-distribution of 1998 from the age distribution and mortality rates of 2008 by back-calculation: e.g. a person who was 30 years old in 2008 must have survived from 1998 until 2008 (using mortality rates $\sigma_{21},...,\sigma_{30}$):

$$N_{30}^{(2008)} = N_{20}^{(1998)} \prod_{i=21}^{30} \mathrm{e}^{-365\sigma_i} \; .$$

More generally, the age distribution at simulation start was given by

$$N_a^{(1998)} = N_{a+10}^{(2008)} \Big/ \prod_{i=a+1}^{a+10} \mathrm{e}^{-365\sigma_i} \; .$$

Birth rates

Making the simplifying assumption that these age-dependent mortality rates do not change during the course of the simulation, the birth rates for the years up to 2008 could be calculated from the demographic distribution of the year 2008 (see Table S1). Neglecting immigrations, at least $N_0^{(2008)}$ = 683,350 babies must have been born in 2008. As $d_0 = 0.3737\%$ of newborn individuals died before the end of their first year, the number of births must have been slightly higher. For the year 2008, the number of individuals $B_0(t)$ between 0 and 1 years of age could be described by the differential equation $dB_0^{(2008)}(t)/dt = \beta^{(2008)} - \sigma_0 B_0^{(2008)}(t)$, where $\beta^{(2008)}$ is the (yet unknown) birth rate and σ_0 is the mortality rate of newborn infants. The solution of this differential equation is $B_0^{(2008)}(t) = \beta^{(2008)}\left(1-\mathrm{e}^{-\sigma_0 \cdot t}\right)/\sigma_0$. At the end of the year (i.e., at $t = 365$), we can set $B_0^{(2008)}(365) = N_0^{(2008)}$ and solve the equation for $\beta^{(2008)}$, obtaining $\beta^{(2008)} = N_0^{2008}\sigma_0 \big/ \left(1-\mathrm{e}^{-365\sigma_0}\right)$. With a similar argument, we obtained the birth rate $\beta^{(2007)}$ from the individuals $N_1^{(2008)}$ who were between one and two years old at the end of 2008, but we had to consider that they further had to survive the year after their 1st birthday: $N_1^{(2008)} = \beta^{(2007)} \dfrac{1-\mathrm{e}^{-365\,\sigma_0}}{\sigma_0} \mathrm{e}^{-365\sigma_1}$. Solving this equation for $\beta^{(2007)}$ yielded $\beta^{(2007)} = N_1^{(2008)}\sigma_0 \big/ \left(\left(1-\mathrm{e}^{-365\sigma_0}\right)\mathrm{e}^{-365\sigma_1}\right)$. More generally, we obtained the birth rates $\beta^{(2008-x)}$ of earlier years from $\beta^{(2008-x)} = \dfrac{N_x^{(2008)}\sigma_0}{\left(1-\mathrm{e}^{-365\sigma_0}\right)\prod_{i=1}^{x}\mathrm{e}^{-365\sigma_i}}$. For years after 2008, we

extrapolated the decreasing trend of birth rates linearly and obtained the daily birth rates $\beta_{2008+x} = 1824.592 - 32.218 * x$.

Comparison with other demographic predictions

The graphs in Figure S1 compare the resulting demographic distribution with the official prediction of the Federal Statistical Office (12th coordinated population projection, scenario: model calculation - migration balance zero). The simulated number of individuals of the years 2010, 2015, and 2020, respectively, are shown as grey bars, the numbers predicted by the Federal Statistical Office are shown as dots. The general agreement between the two prediction models is very good, but the simulated values predict a slightly smaller number of children and smaller numbers for some of the age classes of elderly persons.

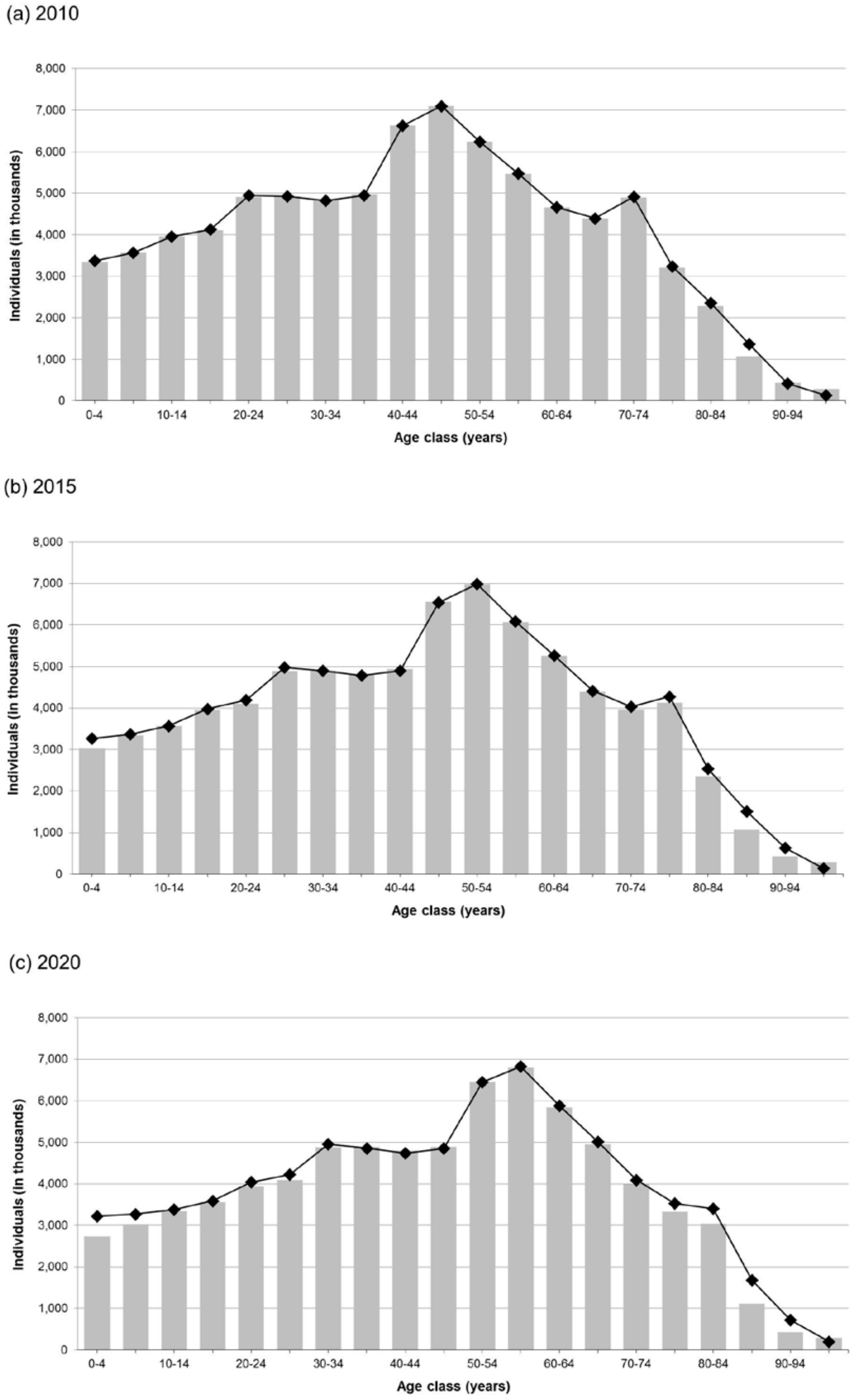

Figure S1. Comparison of the simulated number of individuals with the number predicted by the Federal Statistical Office (Statistisches Bundesamt 2009).

Undiscounted and discounted life expectancy

To calculate the number of life years lost due to influenza, we had to calculate the remaining life expectancy E_a of a person who is a years old. To obtain this, we took the current age a of the individual and added up the fractions of individuals of this age who would survive another year, another two years, and so on: $E_a = \sum_{i=0}^{\infty} \prod_{k=1}^{i} \left(1 - d_{a+k}\right)$. For economic analyses, we also needed the "discounted life expectancy" of an individual. Using an annual discount rate $d_€$ of 3%, we obtained the discounted life expectancy of a person who is a years old as $\tilde{E}_a = \sum_{i=0}^{\infty} \left(\frac{1}{1-d_€}\right)^i \prod_{k=1}^{i} \left(1 - d_{a+k}\right)$. Lost life years which would have been spent i years in the future, were discounted with a factor $\left(\frac{1}{1+d_€}\right)^i$, as events were assumed to "weigh less" when they occurred in a distant future (for the discounting process, we used the start of the intervention period as time zero).

2 Vaccination

Vaccination scenarios and vaccination coverage

During the run-in phase which starts on September 1st, 1998 (scenario 0), an age-dependent percentage of the population was annually vaccinated with TIV. On September 1st, 2012, the intervention phase of the simulations began: in scenario 1, the run-in vaccination was continued without any change whereas in scenario 2, the TIV vaccination of children from 2 to 17 years of age was replaced by LAIV whereas the other age groups continued receiving TIV as before. In the following years, the LAIV vaccination coverage was gradually increased in scenario 2 until it reached its maximum value where it was kept during the remaining years of the intervention phase. As we also varied TIV immunisation in some of our sensitivity analyses, we give the full model description here, which allows for increasing LAIV and TIV immunisation in scenario 2.

Let $c^{(0)}_{TIV,\mathrm{g},a}$ and $c^{(1)}_{TIV,\mathrm{g},a}$ be the TIV coverage of age class a and risk status g in scenario 0 and 1, respectively, whereby g either is "n" (normal risk) or "r" (elevated risk). The TIV coverage of risk group g and age class a at time t in scenario 2 was then given by

$$c^{(2)}_{TIV,\mathrm{g},a}(t) = \begin{cases} c^{(2)}_{TIV,start,\mathrm{g,a}} + \left(c^{(2)}_{TIV,end,\mathrm{g},a} - c^{(2)}_{TIV,start,\mathrm{g},a}\right) \cdot \left(y(t) - 2012\right)/3 & if \;\; 2012 \le y(t) < 2014 \\ c^{(2)}_{TIV,end,\mathrm{g},a} & if \;\; y(t) \ge 2014 \end{cases}$$

whereby $c^{(2)}_{TIV,start,\mathrm{g},\mathrm{a}}$ was the initial TIV coverage in 2008 and $c^{(2)}_{TIV,end,g,a}$ was the final vaccination coverage after 3 years. The LAIV vaccination coverage of risk group g and age class a at time t in scenario 2 was given by

$$c^{(2)}_{LAIV,\mathrm{g},a}(t) = \begin{cases} c^{(2)}_{LAIV,start,\mathrm{g},\mathrm{a}} + \left(c^{(2)}_{LAIV,end,\mathrm{g},a} - c^{(2)}_{LAIV,start,\mathrm{g},a}\right)\cdot\left(y(t) - 2012\right)/3 & if\ \ 2012 \le y(t) < 2014 \\ c^{(2)}_{LAIV,end,\mathrm{g},a} & if\ \ y(t) \ge 2014 \end{cases}$$

whereby $c^{(2)}_{LAIV,start,\mathrm{g},\mathrm{a}}$ is the initial LAIV coverage in 2008 and $c^{(2)}_{LAIV,end,g,a}$ was the final vaccination coverage after 3 years. The vaccination coverage was set for each year; $y(t)$ is the year corresponding with the simulation time t (with $1998 \le y(t) \le 2022$).

Annual vaccination

Vaccinations were performed annually from 1 October (t_{start}) to 30 November (t_{end}). In order not to vaccinate individuals twice within one year, vaccinated individuals were moved from their original compartment to a different one which was marked with an asterisk to indicate that they could not be vaccinated again in the current simulation year. At the end of the simulation year, all individuals were again declared eligible for vaccination by moving vaccinated individuals back to the class of unvaccinated ones.

Vaccination rates

To simplify the description of the vaccination rates, we defined $d(t)$ as the day of the simulation year which corresponds with simulation time t (for 1 September 1st, we set $d(t)=1$). The vaccination rate $\varphi(t)$ was calculated such that the daily number of vaccinations in the vaccination interval remained approximately constant during the annual vaccination period and that a given percentage of individuals would finally be vaccinated (due to deaths during the vaccination period, the achieved number of vaccinations slightly deviated from the goal). In order to facilitate the description of the differential equations, the vaccination scenario are shown as superscript sc: $\varphi^{(sc)}_{LAIV,\mathrm{g},a}(t)$ and $\varphi^{(sc)}_{TIV,\mathrm{g},a}(t)$ denote the vaccination rates of individuals of age a at time t, whereby sc denotes the vaccination scenario (0, 1 or 2).
TIV vaccination rate in the run-in phase (scenario 0):

$$\varphi^{(0)}_{TIV,\mathrm{g},a}(t) = \begin{cases} \dfrac{c^{(0)}_{TIV,\mathrm{g},a}}{\left(t_{end} - t_{start}\right) - \left(d(t) - t_{start}\right)c^{(0)}_{TIV,\mathrm{g},a}} & if\ t_{start} \le d(t) \le t_{end} \\ 0 & otherwise \end{cases}$$

TIV vaccination rate in intervention scenario 1:

$$\varphi^{(1)}_{TIV,g,a}(t)=\begin{cases}\dfrac{c^{(1)}_{TIV,g,a}}{(t_{end}-t_{start})-(d(t)-t_{start})c^{(1)}_{TIV,g,a}} & if\ t_{start}\le d(t)\le t_{end}\\ 0 & otherwise\end{cases}$$

TIV vaccination rate in intervention scenario 2:

$$\varphi^{(2)}_{TIV,g,a}(t)=\begin{cases}\dfrac{c^{(2)}_{TIV,g,a}(t)}{(t_{end}-t_{start})-(d(t)-t_{start})\left(c^{(2)}_{LAIV,g,a}(t)+c^{(2)}_{TIV,g,a}(t)\right)} & if\ t_{start}\le d(t)\le t_{end}\\ 0 & otherwise\end{cases}$$

LAIV vaccination rate in intervention scenario 2:

$$\varphi^{(2)}_{LAIV,g,a}(t)=\begin{cases}\dfrac{c^{(2)}_{LAIV,g,a}(t)}{(t_{ende}-t_{start})-(d(t)-t_{start})\left(c^{(2)}_{LAIV,g,a}(t)+c^{(2)}_{TIV,g,a}(t)\right)} & if\ t_{start}\le d(t)\le t_{end}\\ 0 & otherwise\end{cases}$$

Duration of immunity after vaccination

The LAIV vaccine efficacy of children was assumed to be 80 % during the first influenza season which follows primary vaccination and 56 % during the second season. As our model assumed that a fraction of vaccinations leads to a protection and that this protection is lost exponentially at rate ξ_{LAIV}, we had to back-calculate the "initial vaccine efficacy" $f_{VE,LAIV,a}$ which would lead to 80 % and 56 %, respectively, in the following two transmission seasons. Assuming that it takes 100 days from vaccination to the peak of the next influenza season, we obtained $f_{VE,LAIV,a}\cdot e^{-100\cdot\xi_{LAIV}}=0.8$, and $f_{VE,LAIV,a}\cdot e^{-(100+365)\cdot\xi_{LAIV}}=0.56$. From these equations, we calculated $\xi_{LAIV}=0.00097719$ per day (corresponding to an average duration of immunity of 2.8 years) and an initial vaccine efficacy $f_{VE,LAIV,a}=88.2\ \%$. For TIV immunisation of children, it was reported that the vaccine efficacy during the first influenza season was 59 %. Assuming that TIV had the same initial efficacy as LAIV (i.e. $f_{VE,TIV,a}=88.2\ \%$), we calculated a loss rate $\xi_{TIV}=0.004022$ from $f_{VE,TIV,a}\cdot e^{-100\cdot\xi_{TIV}}=0.59$ (corresponding to an average duration of immunity of 0.7 years). Extrapolating this to the second season after vaccination yielded a remaining TIV vaccine efficacy of $f_{VE,TIV,a}\cdot e^{-(100+365)\cdot\xi_{TIV}}=13.6\%$.

3 Contact structure and infection transmission

Contact matrix

We used the POLYMOD matrix for Germany (Mossong et al. 2008) to calculate effective contact rates κ_{a_S,a_I}. As the original POLYMOD matrix was defined for 10 age groups of 5 years (see Table S2), we first have to extend this matrix to accommodate 96 cohorts: (a) We duplicated the lines and columns of the POLYMOD matrix so that it became a 96 x 96 matrix. (b) We then multiplied each line i of the resulting matrix with $N_i^{(2008)} \big/ \left\langle N_i^{(2008)} \right\rangle$ to obtain the Extended POLYMOD Matrix ($N_i^{(2008)}$ was the 2008 population size of the cohort i; $\left\langle N_i^{(2008)} \right\rangle$ was the sum of the cohorts of the original POLYMOD age class to which cohort i belongs; e.g., for $i=12$, it is $N_{12}^{(2008)} \big/ \left\langle N_{12}^{(2008)} \right\rangle = N_{12}^{(2008)} \big/ \sum_{k=10}^{14} N_k^{(2008)}$). In the next step, we transformed the obtained contacts into "contacts which are efficiently close for transmission". This was obtained by first constructing the Next Generation Matrix by multiplying each element of the Extended POLYMOD Matrix with the duration of infectiousness D_I, and then calculating the largest real eigenvalue $e_{\max}$ of the this matrix. Using the value of the basic reproduction number R_0 to calibrate transmission, we obtained the correction factor $R_0 / e_{\max}$ with which the Extended POLYMOD Matrix had to be multiplied to obtain the matrix of effective contact rates κ_{a_S,a_I}.

Table S2. POLYMOD contact matrix for Germany (Mossong et al. 2008).

Age of the contact	Age of the participant														
	0-04	05-09	10-14	15-19	20-24	25-29	30-34	35-39	40-44	45-49	50-54	55-59	60-64	65-69	≥70
00-04	1.90	0.36	0.09	0.04	0.08	0.27	0.46	0.20	0.20	0.09	0.19	0.07	0.10	0.03	0.07
05-09	0.81	2.25	0.35	0.18	0.03	0.17	0.26	0.49	0.23	0.09	0.20	0.11	0.14	0.11	0.27
10-14	0.34	0.61	3.58	0.69	0.12	0.16	0.14	0.62	0.67	0.35	0.16	0.07	0.07	0.18	0.34
15-19	0.42	0.42	0.93	4.19	0.90	0.28	0.19	0.46	1.11	1.21	0.27	0.26	0.11	0.15	0.49
20-24	0.56	0.24	0.25	1.29	2.59	1.43	0.44	0.22	0.57	0.45	0.47	0.33	0.19	0.15	0.30
25-29	0.62	0.43	0.25	0.36	1.14	1.79	0.98	0.30	0.38	0.57	0.60	0.45	0.32	0.19	0.24
30-34	1.20	0.66	0.34	0.22	0.45	1.09	1.49	0.74	0.57	0.59	0.54	0.61	0.31	0.32	0.41
35-39	0.87	0.77	0.65	0.60	0.45	0.77	1.18	1.43	0.95	0.70	0.58	0.57	0.36	0.42	0.50
40-44	0.49	0.72	0.97	0.69	0.47	0.60	0.98	1.03	1.23	1.05	0.71	0.59	0.49	0.62	0.52
45-49	0.40	0.38	0.44	0.68	0.63	0.77	0.32	0.59	0.93	1.23	0.99	0.62	0.44	0.38	0.37
50-54	0.38	0.23	0.37	0.39	0.41	0.59	0.67	0.36	0.47	0.82	1.18	0.79	0.40	0.45	0.38
55-59	0.26	0.26	0.15	0.26	0.26	0.59	0.35	0.28	0.22	0.41	0.79	1.12	0.75	0.35	0.31
60-64	0.27	0.22	0.19	0.12	0.11	0.49	0.42	0.36	0.32	0.28	0.42	0.54	0.85	0.80	0.43
65-69	0.19	0.18	0.19	0.15	0.08	0.23	0.19	0.30	0.20	0.15	0.18	0.30	0.52	0.91	0.44
≥70	0.38	0.63	0.24	0.14	0.19	0.28	0.21	0.22	0.40	0.26	0.28	0.34	0.37	1.30	0.76

Force of infection

In order to obtain the force of infection for individuals of age a_S, we had to multiply the effective contact rates κ_{a_S,a_I} with the number of infectious individuals I_{a_I} of the different age classes a_I and to divide the product by the total number of individuals of age a_S (for this, we used the size $N_{a_S}^{(y(t)-1)}$ of age class a_S which was obtained at the end of the preceding year). As the transmission was assumed to fluctuate seasonally, the product had to be multiplied by the seasonal factor $1+z\ \cos(2\pi(t-t_{\max})/365)$ (Vynnycky et al. 2008). As each simulation year started on September 1st, the transmission probability reaches its maximum on $t_{\max}$ =112 (i.e., on December 21st), where it is z = 43% larger than the baseline value. Additional to the possibility of being infected by an influenza case “within the country”, people were assumed to be exposed to an “outside infection rate” $\alpha = 0.001$ per year throughout the simulation. Combining all these factors lead to the following force of infection for susceptible individuals of age a_S at time t:

$$\lambda_{a_S}(t) = \alpha + \left(1+z\ \cos\left(2\pi\frac{t-t_{\max}}{365}\right)\right)\left(\sum_{a_I=0}^{95}\kappa_{a_S,a_I}\left(I_{\mathrm{n},a_I}(t)+I_{\mathrm{r},a_I}(t)+I^*_{\mathrm{n},a_I}(t)+I^*_{\mathrm{r},a_I}(t)\right)\right)/N_{a_S}^{(y(t)-1)}$$

with $\left(I_{\mathrm{n},a_I}(t)+I_{\mathrm{r},a_I}(t)+I^*_{\mathrm{n},a_I}(t)+I^*_{\mathrm{r},a_I}(t)\right)$ being the sum of all infectious individuals of age a_I irrespective of risk or vaccination status.

4 Transmission model

Variables

M	number of individuals who are protected by maternal antibodies
S	number of susceptible individuals
L	number of infected individuals (latent phase)
I	number of infectious individuals
R	number of naturally immunised individuals
V_{LAIV}	number of individuals who are immune due to LAIV immunisation
V_{TIV}	number of individuals who are immune due to TIV immunisation

C_I	total number of infections
C_{LAIV}	total number of LAIV immunisation
C_{TIV}	total number of TIV immunisation

x_a index a indicates the age class

x_g index g indicates the risk group:

$g = n$ stands for “normal risk”, $g = r$ for “elevated risk”

x^* the asterisk marks people who have been vaccinated in the current simulation year

Parameters which allow for modelling “leaky vaccines”

For the sensitivity analysis in which we assess the herd immunity conferred by vaccination, we need additional parameters:

p_R probability that a person with naturally acquired immunity can be infected

p_{TIV} probability that a person with immunity acquired by TIV vaccination can be infected

p_{LAIV} probability that a person with immunity acquired by LAIV vaccination can be infected

Whereas the infection probability of immune individuals is 0% in the baseline setting of our simulations, it is changed for vaccine-induced immunity to 100% in a sensitivity analysis. Unlike after infection of previously susceptible individuals, it is further assumed that immune individuals do not develop disease upon infection (i.e. the vaccine only protects against disease, but not against infection).

Initial values

Initial number of susceptible individuals: $S_{n,a}(0) = (1 - f_R)N_a\ (1 - r_a)$

$$S_{r,a}(0) = (1 - f_R)N_a\ r_a$$

Initial number of immune individuals: $R_{n,a}(0) = f_R N_a\ (1 - r_a)$

$$R_{r,a}(0) = f_R N_a\ r_a$$

All other variables were initially set to zero.

Dynamic model

Children with maternal protection (age classes 0 and 1 only)

$$\frac{dM_{g,a}}{dt} = -\left(\varphi^{(sc)}_{LAIV,g,a}(t) + \varphi^{(sc)}_{TIV,g,a}(t) + \mu + \sigma_a\right)M_{g,a} + \begin{cases} \beta(t)m & if \quad (g = n \quad and \quad a = 0) \\ 0 & else \end{cases}$$

$$\frac{dM^*_{\mathrm{g},a}}{dt} = \left(\varphi^{(sc)}_{LAIV,\mathrm{g},a}(t) + \varphi^{(sc)}_{TIV,\mathrm{g},a}(t)\right)M_{\mathrm{g},a} - (\mu + \sigma_0)M^*_{\mathrm{g},a}$$

Susceptible individuals

$$\begin{aligned} \frac{dS_{\mathrm{g},a}}{dt} = {} & \xi_R R_{\mathrm{g},a} + \xi_{LAIV} V_{LAIV,\mathrm{g},a} + \xi_{other} V_{TIV,\mathrm{g},a} - \left(\varphi^{(sc)}_{LAIV,\mathrm{g},a}(t) + \varphi^{(sc)}_{TIV,\mathrm{g},a}(t) + \lambda_a(t) + \sigma_a\right)S_{\mathrm{g},a} \\ & + \begin{cases} \beta(t)(1-m) & if \quad (\mathrm{g} = n \;\; and \;\; a = 0) \\ 0 & else \end{cases} \\ & + \begin{cases} \mu M_{\mathrm{g},a} & if \quad a < 2 \\ 0 & else \end{cases} \end{aligned}$$

$$\begin{aligned} \frac{dS^*_{\mathrm{g},a}}{dt} = {} & \xi_R R^*_{\mathrm{g},a} + \xi_{LAIV} V^*_{LAIV,\mathrm{g},a} + \xi_{TIV} V^*_{TIV,\mathrm{g},a} \\ & + \left(\left(1 - f_{VE,LAIV,a}\right)\varphi^{(sc)}_{LAIV,\mathrm{g},a}(t) + \left(1 - f_{VE,TIV,a}\right)\varphi^{(sc)}_{TIV,\mathrm{g},a}(t)\right)S_{\mathrm{g},a} - \left(\lambda_a(t) + \sigma_a\right)S^*_{\mathrm{g},a} \\ & + \begin{cases} \mu M^*_{\mathrm{g},a} & if \quad a < 2 \\ 0 & else \end{cases} \end{aligned}$$

Infected individuals in the latent period

$$\frac{dL_{\mathrm{g},a}}{dt} = \lambda_a(t)\left(S_{\mathrm{g},a} + p_{LAIV} V_{\mathrm{LAIV},\mathrm{g},a} + p_{TIV} V_{\mathrm{TIV},\mathrm{g},a} + p_R R_{\mathrm{g},a}\right) - \left(\varphi^{(sc)}_{LAIV,\mathrm{g},a}(t) + \varphi^{(sc)}_{TIV,\mathrm{g},a}(t) + \delta + \sigma_a\right)L_{\mathrm{g},a}$$

$$\frac{dL^*_{\mathrm{g},a}}{dt} = \lambda_a(t)\left(S^*_{\mathrm{g},a} + p_{LAIV} V^*_{\mathrm{LAIV},\mathrm{g},a} + p_{TIV} V^*_{\mathrm{TIV},\mathrm{g},a} + p_R R^*_{\mathrm{g},a}\right) + \left(\varphi^{(sc)}_{LAIV,\mathrm{g},a}(t) + \varphi^{(sc)}_{TIV,\mathrm{g},a}(t)\right)L_{\mathrm{g},a} - (\delta + \sigma_a)L^*_{\mathrm{g},a}$$

Infectious individuals

$$\frac{dI_{\mathrm{g},a}}{dt} = \delta L_{\mathrm{g},a} - \left(\varphi^{(sc)}_{LAIV,\mathrm{g},a}(t) + \varphi^{(sc)}_{TIV,\mathrm{g},a}(t) + \gamma + \sigma_a\right)I_{\mathrm{g},a}$$

$$\frac{dI^*_{\mathrm{g},a}}{dt} = \delta L^*_{\mathrm{g},a} + \left(\varphi^{(sc)}_{LAIV,\mathrm{g},a}(t) + \varphi^{(sc)}_{TIV,\mathrm{g},a}(t)\right)I_{\mathrm{g},a} - (\gamma + \sigma_a)I^*_{\mathrm{g},a}$$

Naturally immunized individuals

$$\frac{dR_{\mathrm{g},a}}{dt} = \gamma I_{\mathrm{g},a} - \left(\varphi^{(sc)}_{LAIV,\mathrm{g},a}(t) + \varphi^{(sc)}_{TIV,\mathrm{g},a}(t) + p_R\lambda_a(t) + \xi_R + \sigma_a\right)R_{\mathrm{g},a}$$

$$\frac{dR^*_{g,a}}{dt} = \gamma I^*_{g,a} + \left(\varphi^{(sc)}_{LAIV,g,a}(t) + \varphi^{(sc)}_{TIV,g,a}(t) + p_R\lambda_a(t)\right)R_{g,a} - (\xi_R + \sigma_a)R^*_{g,a}$$

Individuals who are immune due to vaccination

$$\frac{dV_{LAIV,g,a}}{dt} = -\left(\varphi^{(sc)}_{LAIV,g,a}(t) + \varphi^{(sc)}_{TIV,g,a}(t) + p_{LAIV}\lambda_a(t) + \xi_{LAIV} + \sigma_a\right)V_{LAIV,g,a}$$

$$\frac{dV^*_{LAIV,g,a}}{dt} = f_{VE,LAIV,a}\varphi^{(sc)}_{LAIV,g,a}(t)S_{g,a} + \left(\varphi^{(sc)}_{LAIV,g,a}(t) + \varphi^{(sc)}_{TIV,g,a}(t) + p_{LAIV}\lambda_a(t)\right)V_{LAIV,g,a} - (\xi_{LAIV} + \sigma_a)V^*_{LAIV,g,a}$$

$$\frac{dV_{TIV,g,a}}{dt} = -\left(\varphi^{(sc)}_{LAIV,g,a}(t) + \varphi^{(sc)}_{TIV,g,a}(t) + p_{TIV}\lambda_a(t) + \xi_{TIV} + \sigma_a\right)V_{TIV,g,a}$$

$$\frac{dV^*_{TIV,g,a}}{dt} = f_{VE,TIV,a}\varphi^{(sc)}_{TIV,g,a}(t)S_{g,a} + \left(\varphi^{(sc)}_{LAIV,g,a}(t) + \varphi^{(sc)}_{TIV,g,a}(t) + p_{TIV}\lambda_a(t)\right)V_{TIV,g,a} - (\xi_{TIV} + \sigma_a)V^*_{TIV,g,a}$$

Cumulative incidence

$$\frac{dC_{I,g,a}}{dt} = \delta\left(L_{g,a} + L^*_{g,a}\right)$$

Cumulative number of vaccinations

$$\frac{dC_{LAIV,g,a}}{dt} = \varphi^{(sc)}_{LAIV,g,a}(t)\left(S_{g,a} + L_{g,a} + I_{g,a} + R_{g,a} + V_{LAIV,g,a} + V_{TIV,g,a} + \begin{cases} M_{g,a} & if \quad a < 2 \\ 0 & else \end{cases}\right)$$

$$\frac{dC_{TIV,g,a}}{dt} = \varphi^{(sc)}_{TIV,g,a}(t)\left(S_{g,a} + L_{g,a} + I_{g,a} + R_{g,a} + V_{LAIV,g,a} + V_{TIV,g,a} + \begin{cases} M_{g,a} & if \quad a < 2 \\ 0 & else \end{cases}\right)$$

Updates at the end of each simulation year

Normal update

The age of the individuals increased by one year at the end of each simulation year and their vaccination label was removed: $X_{g,a}(new) \leftarrow X_{g,a-1}(old) + X^*_{g,a-1}(old)$, whereby X stands for M, S, L, I, R, V_{LAIV} and V_{TIV}; the number of vaccinated individuals was set to zero: $X^*_{g,a}(new) \leftarrow 0$.

Exception 1: youngest and oldest age class

At the beginning of each new year, the youngest age class was still empty (it was filled with newborns during the year): $X_{g,0}(new) \leftarrow 0$. The oldest age class kept accumulating individuals:
$X_{g,95}(new) \leftarrow X_{g,94}(old) + X_{g,95}(old)$.

Exception 2: individuals with maternal protection

Maternal protection was assumed to last for at most two years. The few individuals who were still maternally protected at the end of two years were added to the number of susceptible individuals:

$$S_{g,2}(new) \leftarrow S_{g,1}(old) + S^{*}_{g,1}(old) + M_{g,1}(old) + M^{*}_{g,1}(old).$$

Exception 3: change in risk status

All children and juveniles up to 17 years of age were assumed to have „normal risk". Accordingly, a change in risk status had to be incorporated at the ageing step from 17 to 18 years. A further increase of risk status was assumed to take place at 45 years. Finally all individuals of at least 60 years were regarded as potential risk patients.

… 18 years old $X_{n,18}(new) \leftarrow (1 - r_{18-44})\left(X_{n,17}(old) + X^{*}_{n,17}(old)\right)$

$$X_{r,18}(new) \leftarrow r_{18-44}\left(X_{n,17}(old) + X^{*}_{n,17}(old)\right)$$

... 45 years old $X_{n,45}(new) \leftarrow \frac{1 - r_{45-59}}{1 - r_{18-44}}\left(X_{n,44}(old) + X^{*}_{n,44}(old)\right)$

$$X_{r,45}(new) \leftarrow X_{r,44}(old) + X^{*}_{r,44}(old) + \frac{r_{45-59} - r_{18-44}}{1 - r_{18-44}}\left(X_{n,44}(old) + X^{*}_{n,44}(old)\right)$$

... 60 years old $X_{n,60}(new) \leftarrow 0$

$$X_{r,60}(new) \leftarrow X_{n,59}(old) + X^{*}_{n,59}(old) + X_{r,59}(old) + X^{*}_{r,59}(old)$$

5 Seasonal waves

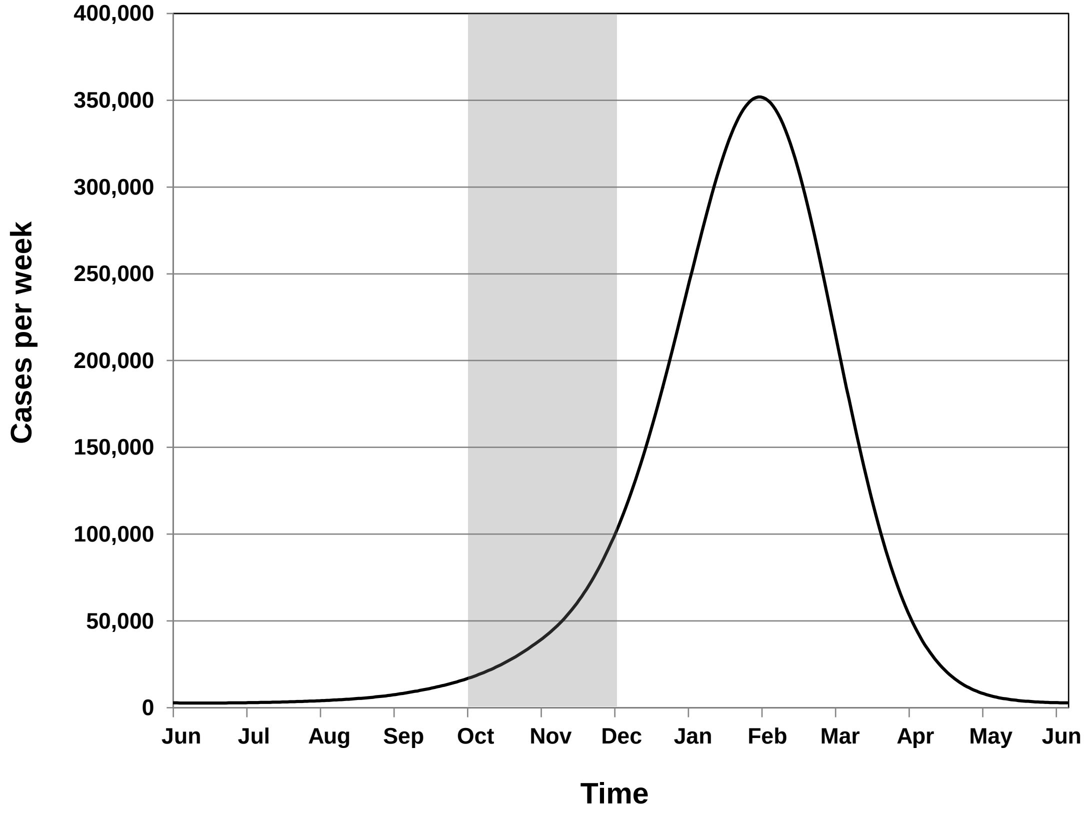

Figure S2. Simulation result of the weekly incidence of influenza cases during the last year of the initialisation period; the annual vaccination period is shown as grey area.

Figure S2 shows the timing of the annual vaccination campaign (grey area) and the course of the subsequent seasonal influenza wave (influenza A and influenza B combined) in the last simulation year of the initialisation period.

References

Mossong J, Hens N, Jit M et al. Social contacts and mixing patterns relevant to the spread of infectious diseases. PLoS Med 2008;5(3):e74.

Statistisches Bundesamt. Bevölkerung Deutschlands bis 2060. 12. koordinierte Bevölkerungsvorausberechnung. 2009. Wiesbaden, Statistisches Bundesamt.

Statistisches Bundesamt. Statistisches Jahrbuch 2010. 2010. Wiesbaden, Statistisches Bundesamt.
